# How Does the Electronic Collection of Patient-Reported Outcomes Improve Patient Engagement in Pharmacy Encounters? A Multi-Method Study

**DOI:** 10.3390/pharmacy13050115

**Published:** 2025-08-27

**Authors:** Bonyan Qudah, Sura AlMahasis, Betty Chewning

**Affiliations:** 1 Department of Medicine, University of Wisconsin, Madison, 1685 Highland Ave, Madison, WI 53705, USA; 2 Division of Social and Administrative Sciences, School of Pharmacy, University of Wisconsin, Madison, 777 Highland Ave., Madison, WI 53705, USA; almahasis@wisc.edu (S.A.); betty.chewning@wisc.edu (B.C.)

**Keywords:** PRO, patient-reported outcomes, community pharmacy, patient-pharmacist communication, patient-centered communication, digital health interventions, medication problems

## Abstract

Routine collection of Patient-Reported Outcomes (PROs) can enhance patient–pharmacist communication and identify medication-related concerns. This study aims to explore the influence of RxTalk™, an electronic PRO tool, on patients’ attributes and the dynamics of communication. Secondary aims include describing patients’ experiences with RxTalk™ and identifying suggestions for improvements. This study is part of a pilot randomized controlled trial in which patients used RxTalk™ in the pharmacy while being observed before they spoke with the pharmacist. Patients’ interactions with pharmacists were tape-recorded and analyzed, and patients were interviewed within one week. We integrated data from RxTalk™, patient observations, taped encounters, and interviews to provide a thicker description of patients’ experiences with RxTalk™ and its impact on their communication. A total of 70% of patients found RxTalk™ easy to use, and 59% perceived RxTalk™ as very useful to extremely useful. Triangulated findings show that RxTalk™ met patients’ social and informational needs, improved their communication skills, and cultivated a sense of privacy to share concerns. Furthermore, RxTalk™ validated the appropriateness of reporting any health concerns, not simply medication concerns. As patients had a positive experience with RxTalk™, pharmacists should consider integrating PRO tools into their daily services to improve patient interactions and quality of care.

## 1. Introduction

Patient-reported outcomes (PROs) are patients’ reports of how they feel, think, and function with regard to their health without the interpretation of clinicians [1]. The collection of PROs in healthcare settings is associated with better symptom monitoring, shared decision-making, improved information exchange, and tailored patient care [2]. Acknowledging the value of routine collection of PROs, regulatory and federal agencies, such as the Agency for Healthcare Quality and Research and The Office of the National Coordinator for Health Information Technology consider research related to patient-generated health data among their top priorities [3,4].

Several interventions have been developed and tested in pharmacies to promote patient engagement and the request for pharmacy services, including question–prompt lists [5,6] and patient-focused communication training [7,8]. However, little research has evaluated how integrating PROs into medication consultations can influence the interaction between patients and pharmacists [9,10,11]. As the landscape of healthcare services has shifted toward higher digitalization and increased personalization of care after the outbreak of coronavirus disease-19, more studies are needed to test and evaluate the influence of digital health interventions on patients’ behavior and attitudes in the pharmacy space [12]. Findings from a systematic review by Wickramasekera et al. suggested that the integration of electronic assessment tools into medical encounters improved the quality of communication between patients and clinicians, especially for psychosocial issues. This ultimately was associated with higher contextualization of care and more effective management of patients’ chronic conditions by clinicians, as they were better informed about patients’ main concerns and treatment priorities [13]. In one study, an app was developed to collect and summarize PROs from patients with cancer, such as insomnia, fatigue, nausea, dyspnea, cognitive problems, and quality of life. The app helped patients set an agenda for the topics that they wanted to discuss with their providers. Mild symptoms (e.g., numbness) were shared by patients, and the app validated the appropriateness of discussing any symptom [14].

In the pharmacy space, using electronic PRO tools holds a significant advantage for both patients and pharmacists. In the fast-paced pharmacy environment, patients can share their medication experiences efficiently and be more involved in the counseling [15]. PRO tools can support pharmacists’ roles through early identification and management of medication-related problems, especially if medication review is not possible. This could eventually lead to improvement in medication adherence and health outcomes. Additionally, they can overcome barriers to effective patient–pharmacist interaction, such as limited time (with less time spent by pharmacists on asking questions), lack of privacy, and pharmacists being perceived as busy or not approachable [16].

Using a randomized controlled design, we explored the impact of RxTalk™, a tablet-based PRO tool, on patient–pharmacist interaction in a community pharmacy setting that wished to try out a new service to improve the quality of pharmacy consultations [17]. After obtaining patients’ consent to join the study, they were randomly assigned to either the intervention group, where they used RxTalk™ in the pharmacy waiting area before receiving their medication consultations, or the control group, where they received standard care and did not use RxTalk™ before their consultation. After intervention patients completed the questions in RxTalk™, the researcher copied key information from RxTalk™ into a paper-based summary sheet, including patients’ PRO scores and types of health and/or medication concerns. Both patients and pharmacists received a copy of the summary sheet and were encouraged to use it during consultation. Their consultation was then audio-recorded. These audio recordings documented that during the consultation with pharmacists, patients who used RxTalk^TM^ were more engaged and expressed significantly more concerns compared to the control group [17].

Since audio recordings of conversations for the purpose of examining patients’ communication behavior in real-world pharmacy consultations are relatively uncommon in the pharmacy literature compared to video recordings [16,18,19], this study offers an additional novel opportunity to explore the mechanism underlying the intervention’s impact on promoting patient engagement and expressing concerns. Guided by communication theories and using multiple data sources, a second set of analyses was performed in this paper to help further our understanding of why RxTalk™ was effective. These analyses could inform the development and refinement of future electronic tools to collect PROs that are easy to use, meet patients’ needs, and help pharmacists provide better care.

Thus, our main objective in this paper is to describe the influence of RxTalk™ on patients’ attributes and the dynamics of communication guided by theoretical frameworks. To better contextualize patients’ experience and understand how RxTalk™ mediated their interactions with pharmacists, we also describe patients’ perceptions of RxTalk™, their responses in the tool, and solicit patients’ suggestions for future improvements.

## 2. Materials and Methods

### 2.1. Intervention Description

RxTalk™ was developed using Qualtrics© 2023 (Qualtrics, Provo, UT, USA). It includes an adaptive questionnaire that tailors questions to each participant based on their responses to previous questions. RxTalk™ assesses the patient’s goals for their pharmacy visit, as well as their health and medication concerns. It also asks about the medication the patient wishes to discuss with their pharmacist, followed by two validated PRO measures related to medication adherence: the Brief Medication Questionnaire (BMQ) [20] and the Merck adherence estimator [21]. We used the abbreviated version of BMQ, which includes four items that ask about how patients took one specific medication in the past week. The 3-item Merck Estimator Adherence measure assesses patients’ likelihood to adhere to their medications, with response categories that range from strongly agree to strongly disagree.

**Figure 1:**
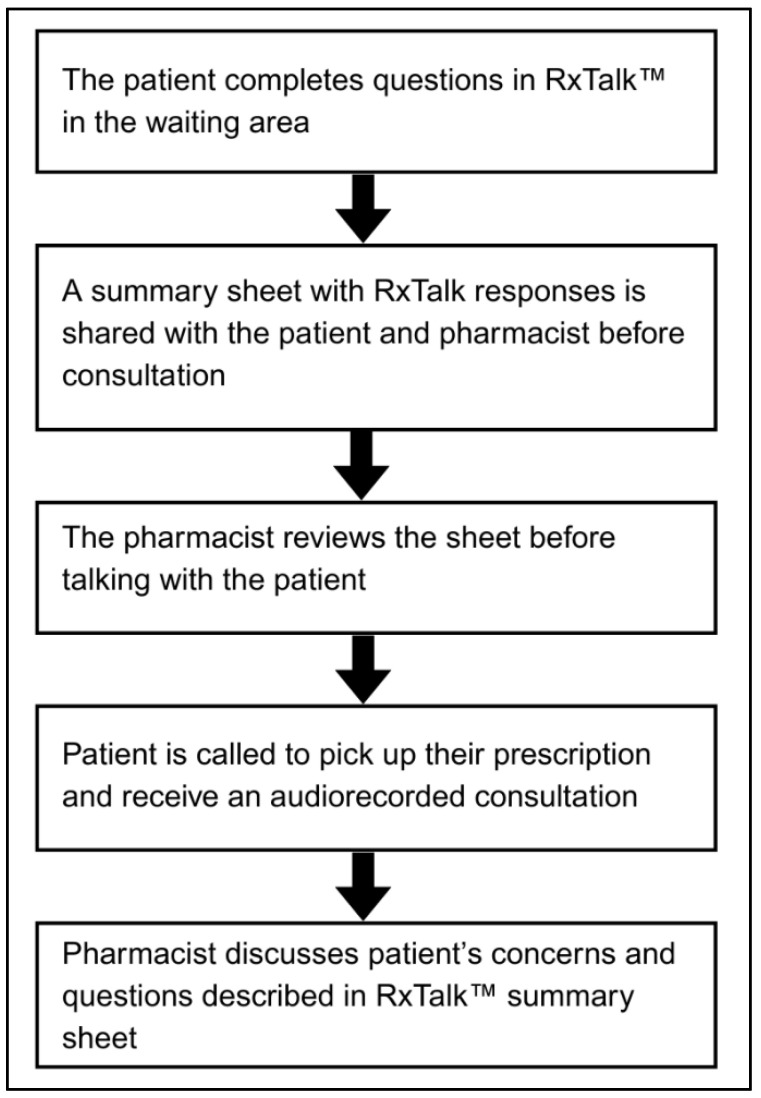
RxTalk™ integration in the pharmacy workflow.

A third PRO measure included in RxTalk™ is the Global Health Rating, a single-item measure that assesses patients’ overall perception of their health, which ranges from poor to excellent [22]. The tool ends with a question that encourages patients to report any additional questions/concerns about their health conditions. For more information on how RxTalk™ was integrated into the pharmacy workflow, refer to Figure 1. A full description of the intervention is published elsewhere [17].

### 2.2. Study Design

This study employed a multi-method approach, utilizing data collected from RxTalk™ users in the intervention group of the pilot randomized controlled trial [17]. Multi-method studies offer numerous advantages for program evaluation, such as providing a comprehensive and richer understanding of the program under investigation, overcoming the weaknesses inherent in single-method studies, and allowing for the triangulation of findings [23]. This study has been approved by the Institutional Review Board of the University of Wisconsin Madison.

### 2.3. Sampling and Recruitment

Patients were recruited from one community pharmacy affiliated with a healthcare system between March and May 2023. Patients were eligible to participate in the study if they were 18 years or older and had at least one chronic illness that required routine prescription medication (s). Caregivers/legal guardians picking up medications for someone else were excluded from the study. Consent was obtained verbally from interested patients after reviewing the study information.

### 2.4. Theoretical Framework and Constructs

**Figure 2:**
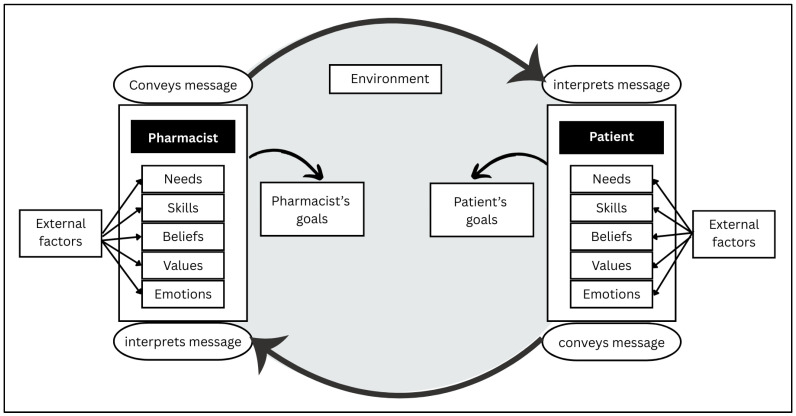
Conceptual framework of patient–pharmacist communication [24].

The Conceptual Framework for Patient–Provider Communication (PPC) guided our data analysis and interpretation to understand how RxTalk™ influenced patient interaction with pharmacists (Figure 2). According to the PPC, communication is a multidimensional process that includes relationship and content and occurs in a complex environment [24]. The success of communication between patients and clinicians depends on enabling both parties to address their goals through conveying and interpreting messages. The model also emphasizes the role of participants’ attributes, such as needs, beliefs, values, skills, and emotions in the communication process. Individuals’ needs include basic human needs and secondary needs (e.g., need for validation). Participants’ values encompass principles and standards that guide their behavior. Skills include participants’ ability to communicate, and beliefs are their perceptions of the world and specifics of a situation. Emotions can have either a negative or a positive valence.

In the context of RxTalk™, we hypothesize that RxTalk™ would provide patients with the appropriate language to help them articulate their health/medication concerns, and it will normalize the discussion around non-adherence through the incorporation of welcoming, non-judgmental verbiage in the tool. Additionally, RxTalk™ would overcome patients’ reluctance to report embarrassing or mild symptoms to pharmacists by validating the appropriateness of reporting any symptom. Finally, we hypothesize that patients’ beliefs of pharmacy consultations would be altered after using RxTalk™ as they realize the range of services pharmacists can provide to improve their medication use experience.

### 2.5. Data Sources and Analysis

Data used in this study were collected and analyzed using the different methods and analytical techniques described in Table 1. All consultations were audio-recorded on the day of enrollment using an encrypted audio recorder placed on the counter. Specifically, 30 consultations were taped in the intervention group. No consultation was excluded based on the content or communication behavior of patients. Two coders (BQ and BC) coded the interactions using a validated tool after establishing interrater reliability [25]. Coding focused on identifying utterances that describe active patient participation, including question-asking, concern expression, and assertive statements. Additionally, we identified the type of health and medication concerns that were shared by patients in the consultation to compare them to patients’ responses in RxTalk™.

Semi-structured interviews were conducted with patients over the phone within one week of enrollment and visit. The interviews were audio-recorded, transcribed verbatim, and deidentified for later analysis by the lead researcher (BQ). Analysis was aided by qualitative analysis software (NVivo, 2020) [26]. Two coders (BQ and SM) coded the interview transcripts using a hybrid approach of inductive and deductive thematic coding. First, the lead researcher conducted a first pass on all interviews to familiarize herself with the data. Then, a few transcripts were read word by word. Statements that described information related to constructs of the PPC were identified. For example, statements that described patients’ attributes, such as emotions (e.g., fear, embarrassment), needs (validation, support, information), or beliefs/values (one should not interrupt pharmacists or only major symptoms should be discussed) and how they were modified by using RxTalk™ were highlighted and coded under the PPC framework. Following the initial coding, the lead researcher coded the remaining transcripts in addition to recoding the former transcripts using the codebook. The codes were later sorted into categories based on how they were related. The second coder analyzed the transcripts independently using the codebook, and weekly meetings were conducted to discuss any discrepancies in coding until a consensus was reached. Inductive content analysis was conducted for the other parts of the interview (data for our secondary aims), which allowed for emerging codes to be included in our data analysis and interpretation.

When available, findings related to patients’ experiences with RxTalk™ were integrated from different data sources to provide a more comprehensive and richer description. For example, patients’ responses to RxTalk™ were compared to the content of taped encounters and interview responses to further explain the changes in patients’ attributes [27].

## 3. Results

### 3.1. Baseline Characteristics and RxTalk™ Data

Thirty patients used RxTalk™ as part of the RCT intervention group. All study patients were white, and the majority were females (73.3%), with a mean (SD) age of 54.9 (15.8). A total of 11 patients had a high school diploma or lower (40.7%), and the remainder had some college or higher (16; 59.3%); education data was missing for 3 patients who were lost to follow-up. Most patients were picking up refill prescriptions (21; 70%) when they enrolled in the study. Half of the patients had hypertension, 43% had chronic pain, 26.7% had mental health conditions, and 20% had diabetes.

In RxTalk™, the patient questionnaire was designed to be adaptive and allowed users to skip questions. Based on that, patients were shown different screens based on their answers. It first identified patients’ medical conditions and then prompted them to select the condition with the most concerns, if any. Among the 30 patients, 22 (73.3%) reported medical condition concerns: 9 (40.9%) feared their condition was uncontrolled, and 7 (31.8%) cited symptoms or side effects concerns. Patients who indicated that they had “No concerns about their medical condition” were asked specifically about medication issues. This stepwise approach helped capture concerns primarily tied to medications. In total, eight (30%) patients struggled with side effects, adherence, or other medication-related problems. Finally, when asked to select a medication to complete PRO measures and discuss with the pharmacist, 70% of patients selected medications taken for more than 6 months. Four patients were excluded from the latter adherence analysis since they selected a new medication. Of the 26 remaining patients, 23.1% were nonadherent to their medications according to the BMQ. However, responses to the Merck Adherence Estimator show that 60% of patients were at moderate to high risk of intentional nonadherence to their medications. Only 6.6% of patients perceived their health as excellent. A full summary of patients’ responses to RxTalk™ can be found in Table 2.

Types of problems observed while patients used RxTalk™ are described in Table 3.

### 3.2. Perceptions of the RxTalk™ Tool

Twenty-seven (90%) of the patients who used RxTalk™ completed the phone interviews. Thus, findings in the following section are based on the triangulation of data for 27 patients.

Patients’ responses to Likert-scale usability questions and relevant quotes from probing questions are summarized in Table 4.

Most interviewed patients (70.4%) perceived RxTalk™ as easy to use compared to only 2 (7.4%) who perceived it as hard (Table 4). During interviews, many patients appreciated the opportunity to answer questions electronically compared to paper and pencil surveys. Being comfortable with technology, availability of researcher support/assistance, and the simplicity of the tool made the experience easy for patients. Moreover, having to wait for a prescription to be filled facilitated the acceptance of RxTalk™. On the other hand, lack of familiarity with tablet computers, difficulty remembering the name of medications, and the need to reflect on health/medication problems before giving an answer affected patients’ perceptions of RxTalk™ usability.

In terms of future use of RxTalk™, only 11% said they were slightly or not likely to use it in the next 3 months. Most patients recognized RxTalk’s potential to facilitate communication with pharmacists and set the agenda for consultations, noting that these features would encourage them to use the tool again in the future. The small number of patients who had low intention to use RxTalk™ in the future cited their preference for direct interaction with pharmacists or no perceived need for using RxTalk™ in the absence of health/medication concerns.

### 3.3. RxTalk™ and the Communication Dynamics

To explore the mechanism by which RxTalk™ influenced patients’ communication behavior of expressing more concerns to pharmacists than did the control group, we examined patients’ goals and expectations, attributes (needs, beliefs, values, skills, and emotions), and perception of the communication context as defined by the PPC framework.

#### 3.3.1. Goals and Expectations

Only three patients (10%) reported in RxTalk™ that one of the goals of their current pharmacy visit was to seek consultations on their medications. Despite this, many patients expressed concerns about their health/medications during their subsequent taped encounters, as seen in our previous paper, where significantly more concerns were expressed by RxTalk™ users than by the control group. This suggests that RxTalk™ not only altered patients’ goals, but it also offered them with additional points to discuss with other members of their healthcare team. For example, one patient (*PT47*) indicated in RxTalk™ that the goal of her pharmacy visit was limited to picking up prescriptions. However, in the interview, she admitted that she had issues with her medications and was satisfied with the pharmacist’s consultation and recommendations.

*“He answered all my questions in a satisfactory manner and gave me a couple of suggestions to discuss with my doctor.”*

*PT47*

#### 3.3.2. Patient Reflection and Skills to Express Concerns

Patients voiced that answering questions in RxTalk™ prompted them to reflect on their health and medication concerns, bringing these issues to the forefront of their minds. Consequently, they felt more prepared and ready to discuss them in consultations. This suggests that RxTalk™ helped equip patients with the skills and the language that they needed to express their concerns. Also, it reduced the amount of information needed to explain their medication and health-related issues.

*“and I thought since you had asked the question, was there one medication that I was concerned about, that kind of then move to the forefront of my mind, that well maybe I can ask him about these things.”*

*PT30*

*“it made it easier than me having to explain it all to the pharmacist after I filled out the survey.”*

*PT24*

#### 3.3.3. Cued Pharmacist to Patient Needs

Patients perceived the summary sheet favorably, articulating that it helped pharmacists become aware of their agenda for that visit and deliver more tailored consultations. This suggests that RxTalk™ improved pharmacists’ skills to identify patients’ highest concerns. This has the potential to streamline consultations and reduce the workload for pharmacists during busy hours.

*“I think it prepares them when they call you up to pick up your medicine to go over the one thing or two things that you were specific in wanting to know.”*

*PT3*

*Interviewer: “So do you feel that there are certain situations that make you more likely to use it in the future versus other situations?”*

*Patient: “When it does get crowded, plus, sometimes, even if it’s not crowded, they do get a lot of calls. There’s a lot of times when someone can’t be there.”*

*PT43*

#### 3.3.4. Needs

Using RxTalk™ fulfilled patients’ need to connect and feel cared for. They appreciated the opportunity to “talk to the pharmacist” after using RxTalk™, which is not common during refill medication pickup.

*“That we got to sit there, talk face to face. Usually, I just go in there and get my medicine and come right back out.”*

*PT26*

In addition to relational needs, the personalized consultation that patients received after using RxTalk™ addressed their informational needs and helped them receive answers to questions that they never thought to ask any of their other healthcare providers.

*“It answered questions that I had for 30 years and just never bothered to ask”*

*PT39*

#### 3.3.5. Beliefs and Values

Patients’ interviews indicate that RxTalk™ lowered the threshold for sharing concerns and validated the appropriateness of discussing any medication-related issue patients might be experiencing, even if they were minor.

*“I think if it would have been a really strong concern, I would have called and talked to them or talked to him at a time when he was, you know, giving me, when I was buying other medicines.”*

*PT30*

In the same vein, the taped encounters demonstrated that issues that are sensitive for patients to raise, such as mental health, were discussed openly in six (20%) of the encounters. The tapes also indicated that patients selected and discussed medications picked up from other pharmacies in three (10%) of the taped encounters, as the pharmacist was heard saying that the medication was not found in the patients’ profiles. This suggests that RxTalk™ helped patients overcome their shared beliefs of only discussing medications that are picked up from that pharmacy [28].

#### 3.3.6. Emotions

A few patients reported that engaging in direct conversations with pharmacists could induce anxiety, particularly if they perceived the pharmacists as busy and not open to interruptions during their work. However, completing the questions in RxTalk™ contributed to alleviating these patients’ concerns and fostered their confidence in voicing their concerns and questions through the tablet.

*“It’s easier to write the answers sometimes on the computer or the tablet because you can be nervous sometimes without asking the pharmacist, especially if they’re busy.”*

*PT5*

#### 3.3.7. Environmental/Contextual Factors

Most of the patients, 19 (70.4%), reported that they had a positive experience completing the RxTalk™ questions in the pharmacy’s waiting room. The remaining patients, 8 (29.6%), had a negative perception of the limited space and inconvenience in the pharmacy.

*“It was probably a pretty good way to do it while I was waiting in order just to fill it out.”*

*PT53*

Since the pharmacy had limited privacy and was located in a close-knit community, patients appreciated the ability to type in their concerns and sensitive information without being heard by others. The privacy feature was especially useful for patients employed at the hospital when the pharmacy was crowded and there was a high risk of losing privacy.

*“I guess the thing, the privacy, part of it. That I’m able to ask that question, and you know it would have been fine if he was able to respond on it that way.”*

*PT30*

It is important to note that no patient reported concerns about losing confidentiality when using RxTalk™ in the interviews, which indicates that patients perceived a low level of risk in using RxTalk™.

### 3.4. Suggestions for Improvement

#### 3.4.1. RxTalk™ Features

Patients gave a few suggestions about improving the design and content of RxTalk™. Comments included adding more instructions, including a text box under health/medication concerns, to provide more contextual information. Additionally, patients suggested integrating a risk assessment system within RxTalk™, which flags patients who require prompt attention by pharmacists and filters out those with no issues or real concerns.

#### 3.4.2. Setting for Using RxTalk™

For future use, only eight patients (29.6%) suggested using RxTalk™ in the same way it was used in the study, in the pharmacy waiting area. Most patients, 14 (51.9%), preferred to use it remotely at home using a link sent to their phones or personal computers. Other suggestions included completing the questions in the pharmacy away from traffic, inside patients’ cars in the parking lot, ahead of medication review appointments, or securing the tablet on a kiosk in the pharmacy for better convenience and privacy.

#### 3.4.3. Feedback from Pharmacists

Seventeen patients (63%) expressed their appreciation for receiving feedback through face-to-face interaction with pharmacists to allow for bidirectional communication. Some patients were also open to other ways of asynchronous communication for receiving pharmacist feedback, such as text messages, email, pharmacy app, or RxTalk™ itself. One patient suggested having a written document with the pharmacist’s recommendation following the consultation. Lastly, a few patients expressed interest in receiving a phone call from their pharmacist to discuss issues that were recalled later.

## 4. Discussion

This study is among the first theoretically driven, in-depth evaluations of the experiences of patients with chronic medical conditions with an electronic PRO tool in a community pharmacy setting.

Our findings suggest that having RxTalk™ collect PRO data about patients was perceived positively, as the tool was quick and straightforward to use, and it gave pharmacists insights into their questions and concerns. The prompt integration of patients’ responses in RxTalk™ into the consultation helped patients see the value of RxTalk™ and how their data was being used by pharmacists, which improved its acceptability. The finding that most patients found RxTalk™ easy and useful becomes even more significant considering that in this rural setting, 40% of the study sample had high school education or less, and many patients expressed a lack of comfort with technology. This suggests that using simple tools, such as survey software loaded onto a touch screen device, can successfully engage patients and monitor medication adherence when health literacy might be a barrier to effective patient–pharmacist communication. However, it is important to note that additional measures should be taken to support patients with different levels of literacy, such as those who cannot remember the names of their medications (by asking patients to bring their medications or medication list before completing the questionnaire) or patients who are not able to read (by adding a read-aloud feature to the tool) [29].

Demonstrating that an intervention is effective [17] is not adequate without exploring how it works, why it works, and under which conditions. The Conceptual Framework for Patient–Provider Communication constructs proved helpful in exploring how RxTalk™ influenced patients’ interaction with pharmacists and their concern expression [30]. Having a written summary for both patient and provider was a key element to cue both the patient and pharmacist during their encounter that a dialogue was expected about the patient’s concerns. Additionally, having RxTalk™ ask a brief set of questions about concerns and behaviors helped patients reflect on and identify their concerns. It gave them language to share these concerns with the pharmacist and validated the appropriateness of discussing long-standing issues and problems not related to the visit. This process of patient reflection and raising pharmacists’ awareness of patients’ issues shifted the conversation from transactional to relational, from the non-medicinal discussion (administrative, logistical) commonly reported in the literature to patients’ driven concerns [31]. Specifically, as RxTalk™ intervention involved both patients and pharmacists, it redistributed the responsibility of guiding and leading the conversation. On one hand, it gave patients an active role in sharing information, which aligns with pharmacists’ expectations of patient responsibility in updating the pharmacist of any changes in medications or the emergence of new health/medication concerns, despite patients historically resisting this unilateral responsibility [28]. On the other hand, by equipping pharmacists with the summary sheet, it helped them lead the discussion by probing, asking clarifying questions, and acknowledging patients’ experiences. This dual engagement offers electronic PRO tools a distinct advantage over other communication interventions, such as question prompt lists (QPLs) that place the responsibility solely on patients to formulate questions and do not offer actionable summaries of patients’ status to pharmacists, such as adherence or beliefs about medications that can foster the discussion and intervention by pharmacists [6]. Future comparative effectiveness studies are needed to explore the usefulness of PRO tools compared to others. Our findings align with other studies that have found a similar positive impact for PRO assessment tools on patient–clinician communication in other settings, such as palliative care [32], general practice [33], and alcohol counseling facility [34], among others.

Pharmacists’ perceptions of RxTalk™ and how it influenced and informed their interactions with patients, compared to standard consultations, was also positive and will be described in detail in a different paper.

Most patients in this study identified concerns in RxTalk™ with medications they had taken for more than 6 months. This underscores the importance of shifting the focus from simply counseling on new prescriptions to including refill prescriptions. Tools such as RxTalk™ could identify which patients have concerns about refill medications. As adherence decreases over time [35], and given that patients rarely raise their concerns about medication-related problems with providers [36], RxTalk™ can help uncover issues through medication use assessment and create more interaction opportunities between patients and pharmacists. By doing so, it can bridge the gap between patients and providers, as supported in this study, and potentially improve health outcomes for patients with chronic medical conditions.

Patients reporting medications refilled from other pharmacy sites in RxTalk™, such as chain pharmacies and mail orders, was an unexpected finding. This highlights that PRO tools can reduce fragmentation in care and contribute to medication reconciliation, especially when implemented in a pharmacy site with strong patient loyalty. This finding will be considered when redesigning RxTalk™ in future studies.

As the management of privacy can be challenging in the busy pharmacy environment [37], RxTalk™ was deemed valuable when patients had concerns about diseases with sensitive connotations, like mental health issues and chronic pain, which were discussed in one-third of the encounters. Given the low frequency of the discussions of these conditions in pharmacy consultations, these findings suggest that RxTalk™ normalized the dialogue about sensitive conditions and provided a safer, private space for sharing the context of these problems [38,39].

The researcher’s assistance was essential for the successful implementation of RxTalk™. While comprehension issues can be resolved by improving the instructions in future iterations of RxTalk™, technical issues need special consideration. Assigning one pharmacy personnel member as a technical support person would encourage patients’ adoption and ensure a seamless experience, especially for older adults and those with limited experience with technology. This aligns with the recommendations from another similar study in clinics, which showed that individual assistance facilitated the uptake and adoption of digital tools [40,41].

### 4.1. Strengths and Limitations

One of the strengths of this study is the utilization of multiple data sources to explore how an interaction with an electronic pre-consultation tool can shape patients’ communication with pharmacists. Using audio recordings to explore patients’ communication behavior was instrumental in furthering our understanding of the nuances of patients’ communication and how it was shaped by the questions they answered before meeting with the pharmacists. So far, the use of audio recording to investigate pharmacy encounters, especially in US-based studies, has been limited to simulated encounters [42,43], while other international studies that have utilized video recordings have focused mainly on pharmacist communication style, and the extent they invited patient participation in the consultation [19].

This study has several limitations. The study was conducted in one rural setting with a relatively small sample, which limits the generalizability of patients’ experience with RxTalk™ to urban and metropolitan settings. However, this was a pilot study testing the usefulness of RxTalk™ in fostering a patient-centered dialogue in the pharmacy. Preliminary findings from this study will be used to test RxTalk™ in a larger, multisite study. Since sampling was not random, it is possible that patients who joined the study were more likely to be receptive to using technology compared to those who did not. However, many patients expressed a lack of comfort with technology at the start of using RxTalk™, which reduces the possibility of this risk. Lastly, future studies should invite patients in the control group to use RxTalk™ as a wait-list control to make an in-depth comparison between the two groups and rule out any possible confounders.

### 4.2. Practice Implications

Pharmacies can adopt several strategies to facilitate the integration of PRO tools in their settings and provide tailored consultation to their patients. The reluctance of some patients to use the tool can be addressed by sending pre-visit text messages to patients to inform them about the PRO service, which can alter their expectations of the visit and its duration. The pharmacy team should also introduce the tool using a carefully crafted message emphasizing the importance of monitoring medication use, efficacy, and side effects [44]. For better efficiency, the pharmacy team can utilize a risk-based approach and target at-risk populations, such as older adults, patients with polypharmacy or multiple comorbidities, and those with refill gaps in the pharmacy dispensing system [45]. Patients who are typically reserved or hesitant in their communication can also benefit from using such tools [46].

Patients’ preference to complete the PRO assessment remotely before the pharmacy visit can be further explored in areas with better internet connectivity to identify the opportunities, challenges, and overall patient experiences. Studies examining the integration of PRO with patient portals (MyChart; Epic Systems) have demonstrated that patients completing PRO assessments in the comfort of their homes was feasible, well accepted, and contributed to higher contextualization of care [41,47]. Additionally, pharmacies can capitalize on patients’ comfort with existing infrastructure, such as their pharmacy app for patient targeting and PRO collection.

## 5. Conclusions

Using RxTalk™ was perceived positively by patients, as it prepared them for the consultation and gave pharmacists insights into their concerns. To expand evidence on the feasibility and acceptability of RxTalk™, future research needs to explore patient experiences in other pharmacy settings and urban locations and include different types of encounters, such as drive-thru visits and videoconferencing consultations.

## Figures and Tables

**Table 1:** Data sources and analytic techniques.

Method	Type of Data Collected	Analytic Technique	Coders
Interviews	Patients’ perceptions of RxTalk™, the consultation, and implementation context were collected using a mix of Likert-scale interview questions and open-ended questions. Further information about the interview guide can be found in the Supplementary Material (Interview guide S1)	Descriptive frequencies and percentages (for Likert-scale questions). Hybrid inductive and deductive thematic analysis for questions related to RxTalk influence on communication. Content analysis for questions related to patients’ experiences with RxTalk™.	Two coders (BQ & SM)
Audio-recorded pharmacy consultations	Names of medications/conditions and medication use barriers discussed during consultations were extracted from the recordings to explore the nature of topics, questions, and concerns raised by patients and pharmacists.	Descriptive frequencies and percentages.	One coder (BQ)
Observations	Problems encountered by patients while using RxTalk™ were recorded using an observation checklist, which classified issues into technical issues and comprehension issues.	Descriptive frequencies and percentages.	One coder (BQ)
Responses in RxTalk™	Goals of the pharmacy visit, medical conditions, health concerns, medication concerns, name of medication for PRO questions, medication dosing schedule, adherence barrier measures (BMQ [20], Merck Adherence Estimator [21], and Global Health scale [22]), additional questions or concerns for pharmacists.	Descriptive frequencies and percentages.	

**Table 2:** Patients’ responses in RxTalk™.

RxTalk™ Questions	Frequency (%) (n = 30)
Goals of pharmacy visit (select all that apply)	
Medication pickup	28 (93.3%)
Get consultation	3 (10%)
Medication review	3 (10%)
Patients’ medical conditions (select all that apply)	
Hypertension	15 (50%)
Chronic pain	13 (43.3%)
High cholesterol	8 (26.7%)
Mental health	8 (26.7%)
Diabetes	6 (20%)
Other *	10 (33.3%)
Conditions with questions/concerns (total) **	
Chronic pain	7 (23.3%)
Diabetes	6 (20%)
Hypertension	5 (16.7%)
Mental health	5 (16.7%)
High cholesterol	2 (6.7%)
Other	7 (23.3%)
No concerns/missing	3 (10%)
Type of medical condition concerns ^†^ (select all that apply)	
Uncontrolled condition	9 (40.9%)
Symptoms or side effects	7 (31.8%)
Following diet	6 (27.3%)
Concerns about medications	8 (36.4%)
Other	2 (9%)
None	1 (4.5%)
Type of medication concerns ^††^ (select all that apply)	
Side effects	5 (62.5%)
Medication not working	2 (25%)
Remembering to take medication	3 (37.5%)
Other	1 (12.5%)
Duration of taking the selected medication	
Less than 1 month	8 (26.7%)
3–6 months	1 (3.3%)
More than 6 months	21 (70%)
Global Health rating	
Excellent	2 (6.7%)
Very good	6 (20%)
Good	11 (36.7%)
Fair	11 (36.7%)
BMQ-Adherent? ^α^	
Yes	20 (76.9%)
No	6 (23.1%)
Merck Adherence Estimator	
Low risk	12 (40%)
Moderate risk	10 (33.3%)
High risk	8 (26.7%)

* Others include asthma, memory problems, prostate, hypothyroidism, migraine, and osteoporosis. ** The numbers reflect all conditions selected by patients after going through the 3 questions about conditions that patients had questions or concerns about. ^†^ Out of the 22 patients who were presented with this question. ^††^ Out of the 9 patients who were presented with this question, there was one missing entry. ^α^ A total of 4 patients were excluded since they selected new prescriptions. Adherence was determined by comparing patients’ responses to BMQ questions to their responses to the question about the regimen recommended by the prescribing clinician. Also, patients reporting missing their medication ≥1 times, in the 4th item of BMQ, were considered nonadherent.

**Table 3:** Problems encountered by patients while using RxTalk™.

Type of Usability Problem	Frequency (%) (n = 30)
Technical difficulties	
Navigating screens	7 (23.3%)
Filling information/clicking on responses	7 (23.3%)
Comprehension problems	
Selecting appropriate answer	11 (36.7%)
Knowing name or spelling of medication	6 (20%)
Understanding the questions/instructions	3 (10%)

**Table 4:** Patients’ perceptions of RxTalk™ elicited during phone interviews.

Perception of RxTalk™ (n = 27)	Frequency (%)	Representative Quotes
Attitude Positive Neutral Negative	22 (77.8%) 3 (11.1%) 2 (7.4%)	Positive *“Very easy to use, not a lot of questions, but it did get to the point of what you were seeking.”PT46* Negative *“ I don’t see the purpose of having to fill that out just to have a consult with the pharmacists” PT21*
Usefulness Extremely useful Very useful Somewhat useful Slightly useful Not at all useful	4 (14.8%) 12 (44.4%) 7 (25.9%) 3 (11.1%) 1 (3.7%)	Useful *“Information wise, you know, I just feel that I get the whole picture of what was going on that way.” PT1* Not that useful *“If I’m going into the pharmacy, I just want to go pick up my medicine. I don’t have to use the iPad in there.” PT7*
Ease of use Easy Hard Neither easy nor hard	19 (70.4%) 2 (7.41%) 6 (22.2%)	Easy *“Everything was pretty clear, and I didn’t have to ask any questions. I understood what the questions were and, you know, how to answer those questions.” PT43* Hard *“I don’t know if it’s so much more using the tablet or if it actually made me kinda have to think of what I’ve been taking for medications to be able to put in there.” PT23*
Intention to use RxTalk™ after 3 months Extremely likely Very likely Somewhat likely Slightly likely Not at all likely	4 (14.8%) 9 (33.3%) 11 (40.7%) 2 (7.4%) 1 (3.7%)	High intention *“I like using technology for stuff like this, I guess.” PT53* Low intention *“If I had any major concerns, the tablet would be helpful. But you know when I go in, just pick up my thyroid med, I wouldn’t necessarily need to use it.” PT48*

## Data Availability

Data can be provided by the corresponding author upon request.

## Supplementary Materials

The following supporting information can be downloaded at https://www.mdpi.com/article/10.3390/pharmacy13050115/s1, File S1: Interview guide S1.
