# Supplementary material for: How Does the Electronic Collection of Patient-Reported Outcomes Improve Patient Engagement in Pharmacy Encounters? A Multi-Method Study
Source: Pharmacy (Basel). 2025 Aug 27;13(5):115. doi: 10.3390/pharmacy13050115 (PMC12452778; doi:10.3390/pharmacy13050115)
Supplement: Supplementary file 1 [file pharmacy-13-00115-s001.zip › pharmacy-3765373-supplementary.pdf]

Phone interview protocol

Please remember that there are no right or wrong answers; we just want your opinion. Your responses will be kept confidential, you can skip any question you don't wish to answer, and only information where your name has been removed will be shared. Do you have any questions before I turn on the recorder and we get started?"

The next few questions will ask about your opinion of the consult that you got after using the tablet computer.

1. How would you describe the conversations you had with your pharmacist when you picked up your last prescription?

Probe: How have your conversations with the pharmacist about medication concerns been affected after using the tablet computer?
Probe: In what ways was the consult different from your previous consults?

2. How responsive was the pharmacist to your needs and concerns?
- Probe: Can you tell me more.

3. How satisfied are you with the counseling that you got after using the tablet computer: not at all satisfied, slightly satisfied, somewhat satisfied, very satisfied, extremely satisfied?
   a. NOT AT ALL SATISFIED
   b. SLIGHTLY SATISFIED
   c. SOMEWHAT SATISFIED
   d. VERY SATISFIED
   e. EXTREMELY SATISFIED
   - Probe: Can you tell me more why you selected "RESPONDENT ANSWER"

4. Do you have health or medication related concerns other than the ones you described in the tablet?
- Probe (IF YES): Did you discuss these concerns with your pharmacist?
- Probe (IF YES): If you don't mind me asking, what stopped you from sharing these concerns with your pharmacists?

5.What other things would you like the pharmacist to know about you so they can provide better care?

6. How do you prefer to get feedback from your pharmacist if the tablet computer was available to you in the future?

7. How private was the space where you received the counseling: not at all private, slightly private, somewhat private, very private, extremely private?

a. NOT AT ALL PRIVATE
b. SLIGHTLY PRIVATE
c. SOMEWHAT PRIVATE
d. VERY PRIVATE
e. EXTREMELY PRIVATE
   - Probe (IF NOT AT ALL PRIVATE-SLIGHTLY PRIVATE): What can the pharmacy do to increase the privacy of the counseling?

8. How noisy was the pharmacy when you received the counseling: not at all noisy, slightly noisy, somewhat noisy, very noisy, extremely noisy?

a. NOT AT ALL NOISY
b. SLIGHTLY NOISY
c. SOMEWHAT NOISY
d. VERY NOISY
e. EXTREMELY NOISY
   - Probe (IF VERY NOSIY- EXTREMELY NOISY): What kind of noises were bothering you?

9. How long have you been using this pharmacy to fill your prescriptions?
__________ (YEARS)

10. In the past two months, did you schedule any medication review appointment at this pharmacy?
(IF YES), when was your medication review appointment?

The rest of the questions will ask about your experience with using the tablet computer at the pharmacy.

11. What were you expecting/hoping to get out of filling your information in the tablet computer?
12. What in particular have you liked or disliked about it?

13. Overall, how useful was the tablet computer in meeting your needs and goals of pharmacy visit: not at all useful, slightly useful, somewhat useful, very useful, extremely useful?

a. NOT AT ALL USEFUL
b. SLIGHTLY USEFUL
c. SOMEWHAT USEFUL
d. VERY USEFUL
e. EXTREMELY USEFUL

- Probe (IF RESPONSE WAS SOMEWHAT USEFUL -EXTREMELY USEFUL): In what ways was it useful to you?
- Probe (IF RESPONSE WAS NOT AT ALL USEFUL -SLIGHTLY USEFUL):
- Can you tell me more?
  What changes do we need to make in the tool to make it useful to you?

14. Would you say answering questions in the tablet computer was easy, hard, or neither easy or hard?

- Probe (EASY): How easy was it to answer the questions: slightly easy, somewhat easy, very easy, extremely easy?
- Probe (EASY): What made the experience easy for you?

- Probe (HARD): How hard was it to answer the questions: slightly hard, somewhat hard, very hard, extremely hard?
- Probe (HARD): What made the experience hard for you?

- Probe (NEITHER EASY OR HARD): Can you tell me more.

15. To what extent did you skip questions? Why is that?

16. Were there any questions that you found too personal or sensitive?
Probe: Can you tell more.

17. Some patients have doubts and concerns when using new technology, did you have any concerns when you used our tool?

- Probe (IF YES): What kind of concerns did you have?
- Probe: (IF YES): What changes should we make to ease your concerns?

18. How likely are you to use the tablet computer if it was available to you after 3 months from now: not at all likely, slightly likely, somewhat likely, very likely, extremely likely?

a. NOT AT ALL LIKELY
b. SLIGHTLY LIKELY
c. SOMEWHAT LIKELY
d. VERY LIKELY
e. EXTREMELY LIKELY
   - Probe: Can you tell more why selected "RESPONDENT ANSWER"?

19. Since our tablet is new in the pharmacy, we are trying to know what the best and most convenient way for patients to use it would be. What do you think of the way you completed the questions?

- Probe: Would you like to complete the questions in a different way like at home, ahead of a medication review appointment, or in the parking lot in your car?
